# Construction of a collection of introgression lines of “Texas” almond DNA fragments in the “Earlygold” peach genetic background

**DOI:** 10.1093/hr/uhac070

**Published:** 2022-03-23

**Authors:** Naveen Kalluri, Octávio Serra, José Manuel Donoso, Roger Picañol, Werner Howad, Iban Eduardo, Pere Arús

**Affiliations:** 1 Centre for Research in Agricultural Genomics (CRAG) CSIC-IRTA-UAB-UB, Campus UAB, Edifici CRAG, Cerdanyola del Vallès (Bellaterra), 08193 Barcelona, Spain; 2 Instituto Nacional de Investigação Agrária e Veterinária, I.P., Banco Português de Germoplasma Vegetal (BPGV), Braga, Portugal; 3 Instituto de Investigaciones Agropecuarias (INIA), Centro Regional de Investigación Rayentué, Av. Salamanca s/n Sector Los Choapinos, Rengo 2940000, Chile; 4 Rijk Zwaan Ibérica S.A. Finca La Marina-PJ Lo Contreras 30395, La Puebla|Cartagena (Murcia), Spain; 5 IRTA, Campus UAB, Edifici CRAG, Cerdanyola del Vallès (Bellaterra), 08193 Barcelona, Spain

## Abstract

Peach [*Prunus persica* L. Batsch] is one of the major temperate fruit tree species, the commercial materials of which have a low level of genetic variability. Almond [*P. dulcis* (Mill) DA Webb], a close relative of peach cultivated for its kernels, has a much higher level of diversity. The species are inter-compatible and often produce fertile hybrids, almond being a possible source of new genes for peach that could provide biotic and abiotic stress tolerance traits. In this paper we describe the development of a collection of peach-almond introgression lines (ILs) having a single fragment of almond (cv. Texas) in the peach background (cv. Earlygold). Lines with few introgressions were selected with markers from successive generations from a “Texas” × “Earlygold” F1 hybrid, initially using a set of SSRs and later with the 18 k peach SNP chip, allowing for the final extraction of 67 lines, 39 with almond heterozygous introgressions covering 99% of the genome, and 28 with homozygous introgressions covering 83% of the genome. As a proof of concept, four major genes and four quantitative characters were examined in the selected ILs giving results generally consistent with previous information on the genetics of these characters. This collection is the first of its kind produced in a woody perennial species and promises to be a valuable tool for genetic analyses, including dissection of quantitative traits, positional cloning, epistasis and as prebreeding material to introgress almond genes of interest into the peach commercial gene pool.

## Introduction

Introgression line (IL), near-isogenic line (NIL) or chromosome segment substitution line (CSSL) collections are sets of lines containing a single fragment of a donor genome in the background of a recurrent genome. These collections consist of tens to a few hundred lines, each with a different introgressed donor fragment, the sum of which cover most, or ideally all, the donor genome [1, 2]. They are usually developed by backcrossing to the recurrent parent in the initial generations, and selfing in the advanced backcross generations, using a set of markers with good genome coverage to identify the individuals of interest.

IL collections serve as valuable tools for genetics and breeding applications, as in the dissection of complex genetic traits [3–6] and the fine mapping and positional cloning of genes or QTLs [7–9]. They are suitable for the study of the effects of specific QTLs in different environments and different genetic backgrounds [10] and the analysis of interallelic and epistatic interactions, as lines with different allelic dosages of one or more QTLs can be created by crossing selected ILs between them or with other lines [11]. Given that ILs are usually constructed with a background of elite commercial lines, they provide an optimal resource to analyze the effects of QTLs of exotic materials in the breeding materials, which are masked in other populations used for genetic analysis by interactions with other loci of exotic origin. This makes IL libraries a direct source of improved materials for plant breeding as well as an invaluable tool for the evaluation and introgression of useful genes from wild or exotic materials in the cultivated gene pool, facilitating the use of the variability stored in exotic materials that can compensate for the loss of diversity resulting from domestication^1^. The first IL collection was produced from a cross between tomato and the wild species *Solanum pennellii* [12]. Since then, IL collections have been extensively developed and used in many model and cultivated species, including most staple crops and horticultural herbaceous species [2]. No examples exist for tree species, due to their long intergeneration periods that makes the process of IL generation extremely long.

Peach was used as the recurrent parent and almond (*P. dulcis*) as donor. Peach and almond are sexually compatible species that originated from a common ancestor in central Asia about 5 Mya [13, 14]. Peach was domesticated in China ~5000 years ago [15], while almond domestication is still unclear, although it probably occurred somewhere between the steppes of central Asia and the eastern Mediterranean shores [16]. Peaches are cultivated for their fleshy mesocarp, as are other *Prunus* stone fruit species such as apricot, cherry, and plum, and almonds are cultivated for their seed: both are of major economic importance.

One of the key biological differences between peach and almond is that peach is self-compatible and has low levels of genetic variability, while almond is self-incompatible with a highly diverse genome [17, 18]. Almond appears to be a good source of new alleles that could provide useful variability conferring adaptation to climate change and disease and pest resistance in peach. Various studies have been undertaken to understand the inheritance of almond variability in the peach background using the offspring of a “Texas” (syn “Texas Prolific”, syn. “Mission”) almond × “Earlygold” peach F1 plant (MB1.37), selfed (the T × E population) and backcrossed to “Earlygold” (the T1E population) [19, 20]. A large backcross progeny was initially used to demonstrate the feasibility of a marker-based method (Marker-Assisted Introgression; MAI) to produce ILs from this interspecific hybrid only two backcross generations after [21]. This was necessary to show that introgression from a distant source was possible within a reasonable timeframe, considering the long intergeneration period of tree species. Among these lines, those carrying a gene for resistance to peach powdery mildew from almond have already been incorporated in the IRTA peach breeding program [22]. Based on the T1E selections, some ILs were extracted and a first collection of individuals with two or three introgressions with the peach cytoplasm in a second backcross with T1E individuals (the E2T set) were developed [21].

The objective of this study was to develop a complete peach-almond IL collection and to describe its main features. Due to the long life and ease of clonal reproduction of *Prunus* trees, two complete sub-collections have been elaborated, one with the almond introgressed fragment in heterozygosis and the other in homozygosis, facilitating the analysis of interallelic interactions. We have tested this collection with a set of characters of simple and complex inheritance of known genetic basis as a proof of concept of its value for genetic analysis. This is a powerful resource to characterize almond diversity in a peach genetic background and will be useful for fruit tree and *Prunus* geneticists and breeders to identify and genetically-dissect valuable traits of almond and to rapidly integrate them in new commercial peach varieties.

**Figure 1:**
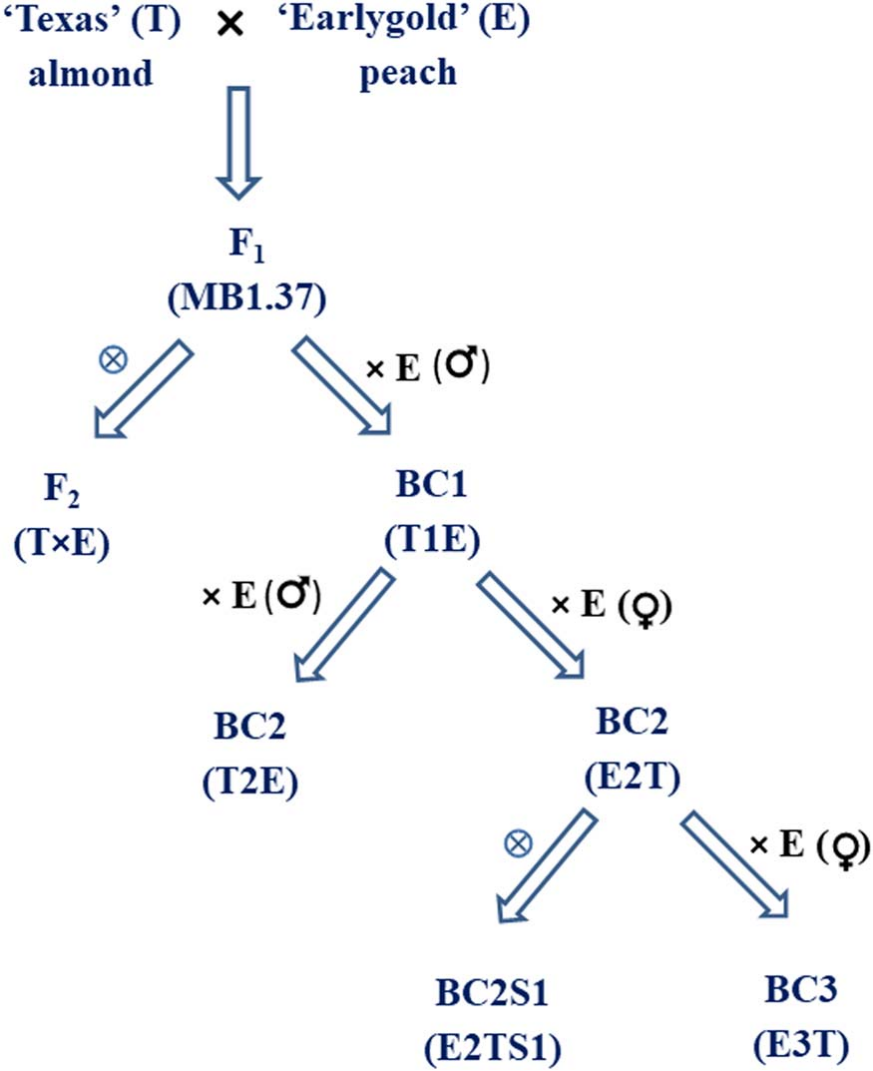
Breeding scheme for the extraction of introgression lines from the almond × peach cross.

## Results

### Development of a peach-almond IL collection

A total of 8467 fruits from different generations of the “Texas” × “Earlygold” cross were obtained from 2011 to 2020 and used to construct this IL collection (Supplementary Table 1, Figure 1). Results to 2015 have been reported earlier [21]. Since that date, 4916 fruits from the offspring of the E2T set, a set of trees from the BC2 generation of the MB1.37 hybrid to “Earlygold” with 2–3 almond introgressions, were used for *in vitro* embryo rescue, giving rise to 1276 seedlings (Supplementary Table 1). Using the 113 SSRs genotyped in the E2T set (Supplementary Table 2 and Supplementary Figure 1), we extracted 146 ILs (97 in heterozygosis and 49 in homozygosis) that, along with the 137 lines (109 in heterozygosis and 28 in homozygosis) previously selected [21], resulted in a full set of 283 ILs covering the complete genome of almond “Texas” in the “Earlygold” background in heterozygosis (206 lines; Supplementary Figure 2) and 94% of the almond genome in homozygosis (77 lines; Supplementary Figure 3). Of the 206 heterozygous ILs, 65 had unique introgressed fragments and in 141 these fragments were in common with other lines (Supplementary Figure 2), whereas for the 77 homozygous ILs, 29 were unique and in the remaining 48 the introgressed fragments were in common with other lines (Supplementary Figure 3). The number of lines covering each linkage group in the heterozygous ILs were G1 (20), G2 (27), G3 (21), G4 (19), G5 (24), G6 (60), G7 (16) and G8 (19). For the 77 homozygous ILs, almond fragments were located on G1 (11), G2 (12), G3 (3), G4 (8), G5 (8), G6 (25), G7 (5) and G8 (5). The missing regions in homozygosis were the ends of G3 and G8 (Supplementary Figure 3). One of these lines (37P18–44) had two introgressed fragments, one in heterozygosis (G1) and another in homozygosis (G2). For all chromosomes in the heterozygous ILs and for chromosomes 2, 4 and 6 in homozygous ILs, at least one of the ILs spanned the whole chromosome distance covered by the markers used.

### Selection of an IL collection with 18 k SNP genotyping

We selected 135 ILs from the total collection to be genotyped for the 18 k SNP chip, including 81 heterozygous ILs (100% almond genome coverage) and 54 homozygous ILs (94% coverage). A total of 6624 SNPs were identified with the appropriate segregations. Markers with “Earlygold” and the MB1.37 hybrid in homozygosis (417) and with missing data in either of them (85) were discarded. The remaining 6122 SNPs nearly covered the entire peach genome (98.4%), with an average density of one SNP every 37 kb. Approximately half (3080) of these SNPs were homozygous for “Earlygold” and heterozygous for MB1.37 and were used to determine the positions of the almond introgressions. The remaining 3042 SNPs, which were heterozygous for “Earlygold and with any genotype for MB1.37, were used to establish the composition of the ‘Earlygold” background.

**Figure 2:**
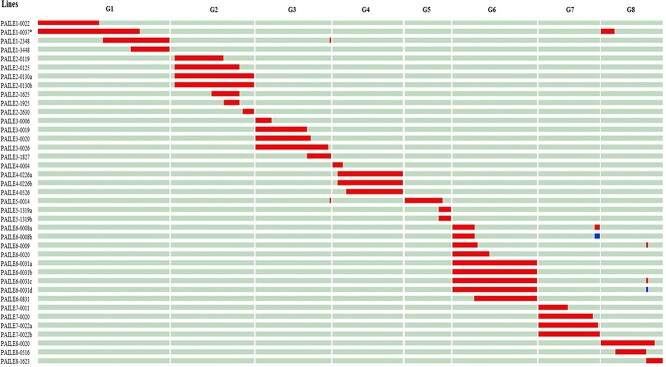
Graphical genotype of the 39 heterozygous ILs of almond (“Texas”) in the peach (“Earlygold”) background based on the 18 k SNP chip and the physical positions of the SNPs. G1 to G8 are the eight linkage groups of *Prunus*.

From the 135 lines analyzed with the 18 k SNP chip, four did not produce reliable results, three did not detect any introgressed fragments and of the remaining 128 lines, 39 were selected for the IL collection in heterozygosis, 38 with a single DNA introgressed fragment from almond and one with two fragments, a major one on G1 and the other on G8. The genome coverage was 99%: including all but one small almond DNA fragment at the proximal end of chromosome 2 (1.35 Mbp) that could not be detected only with the SSRs used (Figure 2, Table 1 and Supplementary Table 3).

**Table 1:** Heterozygous and homozygous IL collections selected with the 18 k SNP chip. Numbers of lines, fragments identified and genome coverage

**ILs HET**									**ILs HOM**						
**Linkage group**	**Mbp total**	**ILs**	**Fragments** *	**Smallest IL fragment (Mbp)**	**Largest IL fragment (Mbp)**	**Average IL fragment (Mbp)**	**Mbp covered**	**Coverage (%)**	**ILs**	**Fragments** *	**Smallest IL fragment (Mbp)**	**Largest IL fragment (Mbp)**	**Average IL fragment (Mbp)**	**Mbp covered**	**Genome cover** **age (%)**
G1	47.8	4	5	14.0	36.9	24.4	47.8	100	3	3	17.0	36.3	24.0	36.3	80
G2	30.4	7	5	4.5	29.1	16.8	29.1	96	5	4	19.2	29.1	23.4	29.1	96
G3	27.4	5	5	6.0	26.4	16.1	27.4	100	2	2	6.0	26.4	16.2	26.4	96
G4	25.8	4	4	4.0	24.3	18.3	25.8	100	2	1	11.1	11.1	11.1	11.1	43
G5	18.5	3	3	6.0	14.0	8.7	18.5	100	3	4	6.0	14.5	11.5	18.5	100
G6	30.8	9	4	8.5	30.8	21.4	30.8	100	8	8	5.1	26.5	14.6	26.5	86
G7	22.4	4	4	11.0	22.4	18.7	22.4	100	2	2	2.2	19.6	10.9	21.8	97
G8	22.6	3	5	6.4	19.5	12.4	22.6	100	3	4	8.5	15.2	11.8	18.4	81
Total	225.7	39	35			17.1	224.4	99	28	28			15.4	188.1	83

* Number of different non-overlapping fragments in which the chromosome is divided by the ILs of the collection

**Figure 3:**
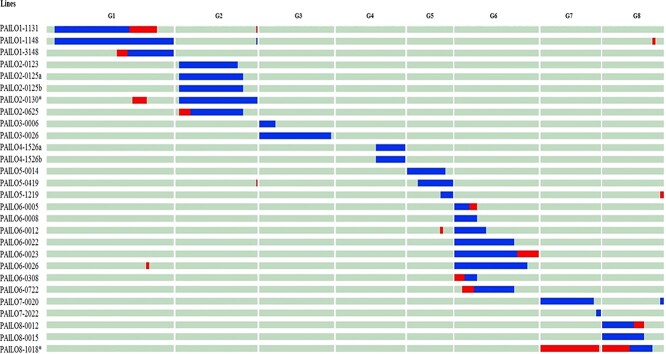
Graphical genotype of the 28 homozygous ILs of almond (“Texas”) in the peach (“Earlygold”) background based on the 18 k SNP chip and the physical positions of the SNPs. Red fragments are heterozygous almond introgressions. G1 to G8 are the eight linkage groups of *Prunus*.

Twenty-eight additional lines with introgressed fragments in homozygosis were also selected, covering 83% of the genome with a large gap on G4 (Figure 3, Table 1 and Supplementary Table 4) and smaller ones on all groups except G5. While most of these plants had a single major fragment in homozygosis, two had two fragments, one in homozygosis and the other in heterozygosis. Nine of the selected lines had part of their introgression in homozygosis and part in heterozygosis, indicating their origin from gametes with recombinations in the almond fragment. These lines would require an additional round of selection in their selfed offspring to obtain completely single-fragment homozygous ILs. The remaining lines genotyped with the SNP chip (60) were discarded as they contained additional almond DNA fragments.

The SNP analysis provided a much more detailed picture of the genome and allowed identification of eight small fragments, <1% of the genome (<2.5 Mbp), that were not detected by the SSRs: one on G1 (0.97 Mbp), G2 (0.82 Mbp), G3 (0.52 Mbp), G5 (1.40 Mbp), and G7 (2.17 Mbp), and three on G8 (0.91, 1.29 and 1.60 Mbp). Their positions are indicated in Figures 2 and 3. They were not considered when selecting or discarding ILs, although one of the homozygous ILs contains only the largest fragment, the one on G7, in homozygosis. Introgressed fragments were generally large (see Table 1), as expected considering the low number of generations used to obtain them, ranging from 4.0–36.9 Mbp (average 17.1 Mbp) in the heterozygous ILs and from 2.2–36.3 Mbp (average 15.4) in the homozygous ILs. Several linkage groups were completely or almost completely (>95%) covered with a single introgression: these were G2, G3, G4, G6, and G7, the two former in both homozygosis and heterozygosis and the rest in heterozygosis.

**Figure 4:**
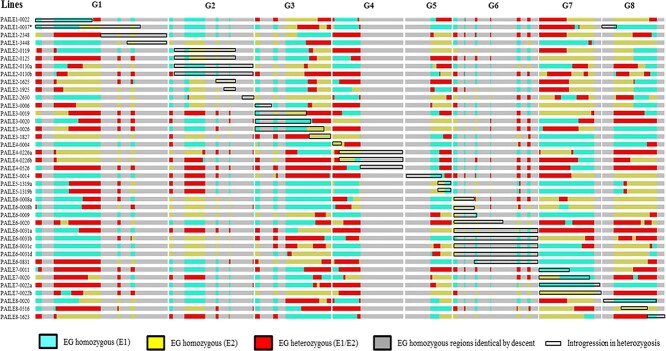
Graphical genotype of the “Earlygold” background in the selected heterozygous ILs using the 18 k SNP chip and the physical positions of the SNPs.

**Figure 5:**
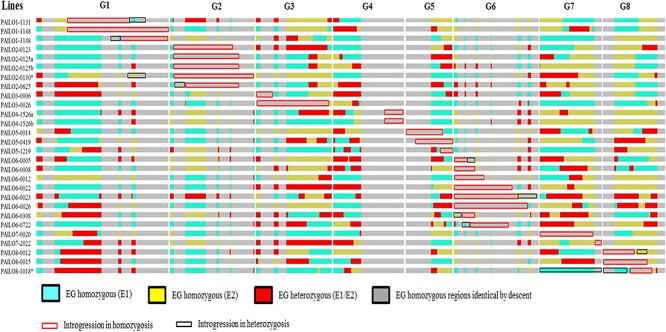
Graphical genotype of the “Earlygold” background in the selected homozygous ILs using the 18 k SNP chip and the physical positions of the SNPs.

Segregation in the “Earlygold” background was also studied with the SNP chip. Results (Figures 4 and 5) show that there were large regions without segregating markers, covering approximately half of the genome (50.5%) as previously observed [19, 23]. The rest of the genome of each IL segregated in large blocks for the two alleles of “Earlygold”. Knowledge of the “Earlygold” genotype for each specific IL is important to incorporate the possible effects of a segregating background in the genetic analysis of trait variability.

### Major genes and QTLs analyzed in the IL collection

The data were obtained from single trees of different ages, some too young to produce fruit, some grown on their own roots and others grafted. For these reasons, and as a preliminary attempt to understand the potential of ILs for genetic analysis, here we studied traits that were determined by major genes and could be analyzed as qualitative, as well as certain quantitative characters previously studied for QTLs in the T × E and T1E populations [20]. Three of the major genes expected to segregate in the ILs, juiciness (*Jui*), blood flesh (*DBF2*) and resistance to powdery mildew (*Vr3*), had the expected phenotypes in all ILs that could be studied, 28, 28 and 63, respectively (Supplementary Table 5). For the two linked genes *Jui* and *DBF2* on chromosome 1, all ILs had the juicy, yellow flesh phenotype except for PAILE1–2348 and PAILE1–3448 that were both non-juicy and red-fleshed. For *Vr3*, only ILs with almond introgressions in the region of chromosome 2 that contains the gene [22] were resistant (PAILE2–0119, PAILE2–0125, PAILE2–0130a, PAILE2–0130b, PAILE2–1625, PAILO2–0123, PAILO2–0125a, PAILO2–0125b, PAILO2–0130*, and PAILE2–0625).

Maturity date (*MD*), a gene located in the central region of chromosome 4 (ref. [24]), was studied in the spring/summer of 2021. This trait has sometimes been scored as qualitative and sometimes as quantitative, depending on the alleles present [20, 23, 24]. In the case of the T × E, T1E and E × E populations (Supplementary Table 6) there are three alleles, one inherited from “Texas” to the MB1.37 hybrid (*T*), and the other two (*E1* and *E2*) from Earlygold, where the *T* allele produces the latest maturity date, while it is intermediate with *E1* and early with *E2*, with gene action predominantly additive. In this case, the average number of Julian days to maturity for ILs without the fragment from almond including *MD* (28 ILs with genotypes involving only alleles *E1* and *E2*), and “Earlygold”, was 167.7 (± 12.4), whilst, as expected, the two ILs (PAILE4-0226b and PAILE4–0526) that contained the late almond *T* allele matured later (the 214^th^ day, more than one month later). However, PAILE4-0226a also containing the *T* allele matured on the 179^th^ day, i.e. within the range of “Earlygold” and the early and intermediate maturing ILs. This can be explained by the “Earlygold” background, where PAILE4-0226b and PAILE4–0526 are *TE1* and have a late phenotype, whereas PAILE4-0226a is *TE2* (see Figure 4), resulting in an individual of intermediate maturity date.

Quantitative measurements were taken for three fruit traits, weight (FW), soluble solid contents (SSC) and titratable acidity (TA) (Supplementary Tables 5 and 7). With the criteria used to consider a significant QTL (see Materials and methods section and Supplementary Table 5), two QTLs were identified for FW, one with a strong increase in fruit weight compared to “Earlygold” in two overlapping homozygous ILs (PAILO6–0005 and PAILO6–0008) with average values of 144 and 148 g/fruit compared to 83 g of “Earlygold” (73–78% weight increase). Another IL in this region, PAILO6–0308, had FW values similar to “Earlygold”, indicating that the QTL was located at the extreme of the chromosome (0–4 Mbp). When looking at heterozygous ILs in this region (PAILE6–0008b, PAILE6–0009, PAILE6-0031a and PAILE6-0031b), we observed an inconsistent pattern with some lines having values similar to “Earlygold” (PAILE6-008b and PAILE6-0031a), and others with significantly lower (PAILE6–0009 and PAILE60031b) values. For SSC, only one highly significant QTL was observed in the proximal end of G6 (PAILO6–0308) with the almond allele producing an increase of the value of this trait, and for TA no QTLs with sufficient effects were identified (Supplementary Table 5).

Petiole length was a variable trait in the crosses between almond and peach studied before [20], where typically almond had a long petiole, peach a short one, intermediate in the hybrid, and the progeny segregated between these two extremes. In the IL collection, all plants had a short petiole like in peach except for PAILE8–0516 and PAILO8–1018 that had a highly significantly longer petiole than ‘Earlygold, similar to that of the interspecific hybrid (Supplementary Tables 5 and 7). One more line (PAILE2–2630), had a significantly shorter petiole than “Earlygold”.

## Discussion

Plant collections adequate for efficient genetic analysis are a key resource for the progress in the genetics and genomics of crop species. Introgression line sets are optimal for the dissection of quantitative traits and to facilitate genetic analysis and gene cloning, particularly in the background of lines of agricultural interest, so avoiding problems related with donor-donor epistatic effects and making possible the detection of subtle pleiotropic effects, usually difficult to identify in other population types such as F2, BC1 or RILs [25]. However, the construction of such populations is especially difficult in woody perennials, as it represents an enormous investment in time and resources, a consequence of the large size of the individuals and the long intergeneration time of these species. In this paper we present a peach-almond introgression collection that we believe is the first to have been produced in a perennial species. One advantage of certain tree crops is that they can be clonally reproduced, which has given us the possibility of constructing two collections, one with 39 ILs that contain introgressed almond fragments in heterozygosity and covers 99% of the almond genome, and another with 28 homozygous ILs with 83% of genome coverage. This continues research begun in 2006 in our lab with the initial objective of finding fast approaches to introgress useful genes from the highly variable *Prunus* wild or cultivated species, almond in this case, to enrich the much narrower variability of cultivated peach. This initial research led to an incomplete first set of introgression lines [21] with partial genome coverage (64% in heterozygosis and 14% in homozygosis). Here, we selected the ILs from the selfed progeny of a set of individuals with a low number of introgressions (2–3), which has considerably simplified our work.

Using a set of SSRs we selected 283 ILs, 135 of which were genotyped in depth with the 18 k SNP chip. SNP results were particularly informative as they increased the marker density more than 50-fold (from an average of one SSR every 2015 kb to one SNP every 37 kb) allowing us to identify and discard plants having more than one introgression. The final collection of 67 individuals with a single introgression (with a few exceptions with two introgressions) could therefore be selected with great certainty, and with a precise position of the boundaries of each almond fragment. The accuracy of this analysis also made possible the identification of several smaller introgressions (<1% of the genome) that were undetected by the SSR markers, and of some missing fragments at the end of certain linkage groups. Our initial expectations were that these fragments would occur only very sporadically because of the relatively low level of recombination of peach chromosomes and specifically of this interspecific cross (1.2 crossovers per chromosome on average [19]), but the fact that we recovered them suggests that a high-density genotyping step before elaborating a final set of ILs is an advisable option.

One aspect to consider in the analysis of this IL collection is that a commercial cultivar (“Earlygold”) was used as a recurrent parent. “Earlygold” is not an inbred line, which has the drawback that segregation for chromosomal fragments heterozygous in this variety may interfere with the interpretation of genes/QTLs from the introgressed almond fragments in the ILs. At the beginning of this project we considered, but discarded, the option of switching to a more homozygous line. The first main reason was that the availability of homozygous lines is scarce in peach, and most of them are either weak individuals, probably as a consequence of inbreeding, or genotypes, such as the Spanish non-melting flesh varieties, that are genetically distant from the major commercial peach gene pool [26]. Secondly, including a new recurrent parent would have delayed the construction of this IL collection by two generations, equivalent to 7–9 years. A third reason is that we expected that the variability detected by the almond genome would often be of a sufficiently different nature and genome position compared to that of peach, as shown in previous results based on the analysis of “Texas” × “Earlygold” progenies [19, 20], making interferences between almond/peach vs. peach/peach easily detectable and interpretable. Additionally, “Earlygold” is heterozygous in less than half of its genome [20, 23], because of the existence of large DNA fragments identical by descent, as in many other modern peach varieties [27], due to the recent history of co-ancestry of modern peach breeding programs [28], so we were only expecting a relatively narrow window of segregation to occur in its offspring. The inheritance analysis of a large set of characters of the F2 of “Earlygold” is available [23], and relevant for this work as it identified a limited number of QTLs. Only one of these, a major QTL located on G4 in the region of *MD* that, in addition to maturity date, partially affects other fruit characters such as fruit weight, soluble solid contents and leaf color at senescence, appears to be of concern when studying fruit or color-related traits. The genotypes for the “Earlygold” background in the IL collection are given in Figures 4 and 5 and may be useful to identify the genotypes of the recurrent parent and consider or discard possible effects of the background on the expression of their character of interest.

Using the plants of the IL collections, we examined the segregation of certain major genes previously described in “Texas” × “Earlygold” progenies, and for four of them (*Jui*, *DBF2*, *Vr3* and *MD*) we observed the expected phenotypes. Other genes that segregate in the IL collection, such as two independent male fertility restorer genes (*Rf1*/*rf1* and *Rf2*/*rf2*) [19] were not expected to produce any phenotypes as all E2T individuals had the peach cytoplasm. The almond fruit (*Alf*) gene that determines the formation of the thick mesocarp [20] typical of peach, would produce only almond fruit types in the individuals homozygous for the recessive almond (*alf*) allele, and the ILs homozygous at the corresponding region of G4 were not available in homozygosity. Other lines having this fragment in homozygosity, along with additional introgressed fragments, were too young to produce fruit. The case of one IL (PAILE4-0226a) with an intermediate maturity date phenotype while it was expected to mature late as it had the almond introgression, can be explained by the interaction of the almond allele with an allele of “Earlygold” that confers early maturity [20, 23], as opposed to other ILs that had the late allele of “Earlygold”. This would be a clear case of segregation in the “Earlygold” background that would result in an unexpected phenotype in an IL. Considering the importance of this region for phenological and fruit related characters, it would be interesting to fix one of the alleles of “Earlygold” at this locus in all lines to have a uniform phenotype for maturity date which may avoid interference with other characters, particularly fruit characters, that may be associated with this region.

Four quantitative characters were studied, three in fruits of some ILs that were already fruiting, and one in the leaf petiole, all with previous inheritance studies and with QTLs identified for some of them (FW and petiole length) in the offspring of crosses involving “Texas” and “Earlygold” [20, 23], the main features of which are summarized in Supplementary Table 6. For one character, fruit weight, a major QTL was previously found at the proximal end of G6 in almond × peach progenies [20], where the allele of almond produced an increase in fruit weight with a high percentage of explained phenotypic variance (*R^2^* = 19–21%). A major QTL for fruit weight was also detected at the same chromosomal region by combining linkage mapping and association studies in a collection of peach genotypes [29]. We found a high increase in fruit weight at this region in the homozygous ILs, but heterozygous ILs had a similar or even significantly lower weight than “Earlygold”, while, based on previous results, an increased size was expected (Supplementary Table 6). A more detailed analysis with uniform and replicated materials is necessary to fully understand the genetics of this especially interesting QTL, as fruit size is a character of great commercial value in peach and the use of the almond allele, alone or in combination with other peach alleles may have an immediate application in breeding. For SSC and TA, for which no QTLs have previously been detected in these materials (Supplementary Table 6), we identified a QTL in the proximal end of G6 for SSC and none for TA. A QTL for SSC at the beginning of chromosome 6 has also been previously reported using a multi-progeny approach that included peach × peach and peach × other species (with almond one of them) [30]. Finally, petiole length was significantly longer in the lines PAILE8–0516 and PAILO8–1018 than in “Earlygold” (43–70% longer) and the other ILs, coinciding with the position of a QTL with large effects (R^2^ = 15–39%) and dominance for the almond allele previously identified [20] for this character. Another IL with petiole length shorter than “Earlygold” was also found in G2, suggesting the existence of alleles that may determine shorter petioles in the species (almond) that has the long petiole phenotype.

The results on the trait analysis reported here were obtained on single plants grown on their own roots and from seedlings obtained in various years (see Supplementary Tables 3 and 4), some of which were still in their juvenile period. While they can be accurate for traits with clear alternative phenotypes that can be scored qualitatively, these conditions are not optimal for the analysis of metric traits. The next step in this research is the production of grafted replicates from the complete IL collection, to be planted in two sites. Once these plants reach the production stage, we will start phenotyping them for the traits that we consider of interest. We also plan to continue the production of new ILs to complete the collection, especially that of homozygous ILs where there are still some gaps. We are also expanding this collection to include ILs with wild crop relatives of peach, particularly *P. davidiana* and *P. mira*. One direction of our current research is to fine map, and eventually clone, the main major genes detected so far in peach × almond crosses. We have already started with the *Vr3* gene that confers resistance to powdery mildew [22], and are advancing with others such as (*DBF2*, *Jui* and *Alf*).

## Conclusion

We report a nearly-complete a collection of ILs that, given the length of the peach intergeneration period, has required a 15-year process of crossing and marker selection along three backcross generations. The collection has been studied in detail with a high-density SNP chip and is ready to be used by members of the scientific community to which we offer any plants in this collection, or the full collection, for research purposes. Apart from its use in enriching the narrow peach variability, and facilitating gene cloning, we believe that this collection is specially adequate for understanding the genetics of two key aspects of the divergence between these two species: the differences between a dry and a fleshy edible fruit from morphological, physiological and metabolic standpoints, and the better adaptation of almonds to abiotic stress, particularly drought, of great importance and urgency considering the menace of climate change.

## Materials and methods

### Plant materials

In previous research, the F1 of the cross between almond “Texas” used as female parent crossed with peach “Earlygold” as pollen donor, named “MB1.37”, was backcrossed to “Earlygold” as male parent, in the winter of 2006, 2007 and 2008 (ref. [21]). A large offspring (N = 1095) was obtained, referred to as the T1E population, and a few (N = 18) individuals, the pre-introgression line (prIL) set, carrying 2–4 almond introgressions were selected with simple-sequence repeat (SSR) markers covering the whole peach genome [21]. The inheritance of various agronomic traits with a group of N = 190 T1E plants was previously analyzed [20], most selected at random from the T1E initial population, but also including those of the prIL set. Due to the cytoplasmic male sterility conferred by the almond cytoplasm detected in the T1E population [19], an additional backcross was performed using “Earlygold” as the female parent (E2T; N = 160), to ensure that the plants obtained were pollen-fertile. The T1E plants used as staminate parents to generate the E2T offspring were chosen to contain a low number of introgressions and to provide full almond genome coverage, including mostly plants from the prIL set. A subset of 37 marker-selected plants of the E2T progeny to contain only two or three almond introgressions, the E2T set, was selected [21]. In this work, the selfed (E2TS1) or backcrossed to “Earlygold” (E3T) progeny of trees from the E2T set were used for this last step of IL extraction, where our objective was to obtain a collection of individuals with a single almond introgression, in both homozygosis and heterozygosis, and covering the full almond genome. The breeding scheme for IL extraction is given in Figure 1.

Plants from parents, hybrid and the T1E generation are kept in the experimental fields of IRTA at Cabrils and Gimenells (Catalonia, Spain) as described before [21]. The E2T set was grown at two different IRTA stations: in Caldes de Montbui grafted to “Garnem” rootstock and in Mollerussa on their own roots. The selected ILs were planted at Caldes de Montbui on their own roots. For the E2T set and ILs the spacing between trees and rows is 2.5 × 4.0 m.

### *In vitro* embryo rescue

As the recurrent parent “Earlygold” is an early maturing variety (May–June), most of the E2T individuals ripened early (June–July). The embryos of these individuals were not completely developed at fruit maturity time and *in vitro* embryo rescue was necessary to be able to germinate all seeds collected. The method used was based on previous work [31]. The fruits were first immersed in a disinfectant solution of water and NaOCl (3.7%), thoroughly washed with water, then opened using a nutcracker to collect the seeds. The seeds were surface sterilized in a solution of 300 ml of NaOCl (3.7%), 700 ml H_2_O and two drops of tween for 10 min, then washed with autoclaved water in a laminar flow hood. Seeds were opened and the embryos extracted and placed in test tubes with a solution of sucrose (30 g/l), Duchefa M0220 (2.46 g/l) and plant agar (8 g/l), with the addition of 10 ml of 100 μM benzyl aminopurine for seeds <5 mm. These were kept in a cold chamber at 4°C for 8–12 weeks or until the onset of radical growth, then transferred to a dark chamber (closed in carton box) at 23°C for one week. After a week in the dark, the embryos were exposed to gradually increasing light conditions. Those plants with well-formed shoots and roots were moved to trays in the greenhouse, and finally to the field.

### Genotyping and introgression line extraction

Genomic DNA was extracted from young leaves using the CTAB method [32] in 96 well plates. Genotyping data from the E2T set were available for 113 SSRs with almost full genome coverage of the peach genome, spanning 212.2 Mbp (359.3 cM), with the average interval between the markers being ~1.9 Mbp (3.2 cM) (Serra et al. 2016; see Supplementary Table 2 and Supplementary Figure 1). Additionally, data from the same SSRs and the 9 k peach SNP chip [33] had been previously obtained [19] in the parents, the F1 and the T1E individuals used for mapping. To select the ILs in the progenies derived from plants of the E2T set we used the same 113 SSRs. The two markers at the extremes of the introgressions of each parent (Supplementary Table 2) were genotyped in the corresponding E2TS1 and E3T progenies, and the individuals with a single introgression in homo- or heterozygosis were selected. For plants having a recombination within one of the introgressed fragments, additional SSRs within this fragment were genotyped until its extremes were identified. Selected individuals were then genotyped with the additional SSRs with coverage of the complete genome.

SSRs were analyzed in the ABI PRISM® 3130xl Genetic Analyzer (Applied Biosystems) by capillary electrophoresis following the PCR amplification method described earlier [26]. For the PCR for SSR amplification, the reaction mix (10 μl) contained 2 μl genomic DNA (200 ng), 1 μl of 10x NH_4_ reaction buffer, 0.3 μl of 50 mM NH_4_ MgCl_2_, 0.2 μl of 10 mM dNTP, 0.2 μl of 10 μM forward primer labeled with fluorochrome (FAM, NED, VIC and PET), 0.2 μl of 10 μM reverse primer, 0.2 μl of 5 U lab Taq and 5.9 μl HPLC water. PCR was performed in a thermocycler (Applied Biosystems, USA) with the following conditions: a single cycle of initial denaturation at 94°C for 1 min, 35 cycles of denaturation at 94°C for 15 s, annealing step between 50 to 65°C for 15 s and an extension step at 72°C for 30 s. One cycle of final elongation was at 72°C for 5 min and at the final step, the PCR product was held at 4°C to complete. For capillary electrophoresis (Applied Biosystems ABI PRISM® 3130xl Genetic Analyzer), 2 μl of PCR product was mixed with 12 μl of formamide, 0.35 μl GeneScan500 LIZ (Applied Biosystems, USA) and denatured at 94°C for 3 min. Allele sizes were identified with the GeneMapper 5.0 software.

A selected set of 135 lines with a single introgression based on their SSR genotype, to cover the largest possible region of the genome, and prioritizing those lines covering whole chromosomes and lines that were already producing fruits, were genotyped with the 18 k Illumina chip [34]. For that, 1000 ng genomic DNA was dried using a speed vacuum and eluted in 20 μl water to a final concentration of 50 ng/μl. DNA purity was checked by Nanodrop absorbance values, with ratios of 1.8 for 260/280 and 2.0–2.2 for 260/230 being considered pure. The samples were genotyped at Fondazione Edmund Mach facility, San Michelle all ‘Adige, Italy.

The raw data from the genotyping platform was processed using Genome Studio Illumina 2.0. The two output files, final report and DNA report from the Genome Studio analysis were used as input files for genetic analysis using the ASSisT software [35], an automatic SNP scoring tool, along with a generated pedigree file and map file. The population was set to germplasm, allowed missing data range to 1.0 and all the other parameters (call rate tolerance, p-value segregation distortion, unexpected genotype threshold per individual and frequency rare allele) set to zero. This way, 41.3% of the SNPs were approved, which was the highest compared to the default and all other settings we tried. Given that peach varieties are known to have large regions identical by descent, chromosomal fragments ≥2.5 Mbp long (approximately 1% of the peach genome) with >3 in total or > 2 consecutive SNPs in heterozygosis, were considered identical by descent and consequently with two identical haplotypes for “Earlygold”. SNP data from IL genotyping have been graphically represented with GGT2 (ref. [36]), using the physical location of the markers in the Lovell peach genome reference sequence v2.0 (ref [37]) as their positional reference.

To identify the ILs obtained individually, we used an internal code to select an initial collection with SSRs (see Supplementary Tables 3 and 4 and Supplementary Figures 2 and 3). For the final IL collection using the data of the 18 k SNP chip, we coined a new terminology: first a group of five letters and a number (the first four letters “PAIL” to indicate Peach-Almond Introgression Line, the fifth letter is E or O indicating heterozygous or homozygous ILs, respectively) and a digit 1–8 corresponding to the chromosome number where the introgression is located. The second part of the IL name is a dash followed by four digits corresponding to the position in Mbp of the two extremes of the introgressed fragments. For example, line PAILO5–1219 is a homozygous peach-almond introgression line located on chromosome 5 and in the region spanning from the 12^th^ to the 19^th^ Mb. A small letter at the end of the IL indicates lines with the same fragment, e.g. PAILE2–0130a and PAILE2–0130b.

### Phenotyping and statistical analysis

The IL collection was evaluated for traits known to have different phenotypes based on the previous information of “Texas” × “Earlygold” progenies [20, 23]. These include several major genes and a few major quantitative trait loci (QTLs). The major genes were: juiciness (*Jui/jui*) as the presence and absence of juice in the fruits at maturity; blood flesh color (*DBF2/dbf2*) as red or yellow flesh color at maturity; maturity date (*MD/md*) as early or late maturing based on the number of Julian days when more than half of the fruits reach ripening stage, estimated on parameters such as fruit firmness and visual observation of fruit skin color change; and powdery mildew resistance (*Vr3/vr3*), scored as resistant or susceptible based on the absence or presence of fungal infection on the leaves. A quantitative leaf character, petiole length (PL), was measured in mm with a ruler in three to six average size leaves of each IL. We phenotyped three additional quantitative fruit traits, taking data from three samples (average of 3–4 fruits per sample) per tree: fruit weight (FW), titratable acidity (TA) and soluble solids content (SSC), measured as described before [20].

Statistical analysis of the data for quantitative traits was performed using the DescTools package of R. The overall data were studied with a one-way analysis of variance, and Dunnett’s test used to compare the mean of each IL with that of the “Earlygold” control. An additional Dunnett’s test was used to integrate the possible effects of traits segregating in the “Earlygold” background in the analysis, comparing the mean of each IL with that “Earlygold” and all the other ILs, excluding those that had the same or part of the same introgressed fragment. Only ILs showing highly significant values (P < 0.01) in both tests were considered as detecting a QTL for the trait studied.

## Supplementary Material

Web_Material_uhac070

## Data Availability

The data underlying this article are available in the article and in its online supplementary material.
