# Supplementary material for: Construction of a collection of introgression lines of “Texas” almond DNA fragments in the “Earlygold” peach genetic background
Source: Hortic Res. 2022 Mar 23;9:uhac070. doi: 10.1093/hr/uhac070 (PMC9157678; doi:10.1093/hr/uhac070)
Supplement: Web_Material_uhac070 [file web_material_uhac070.zip › Peach-almond ILs R Supplementary Materials.docx]

**Supplementary Table 1**. Introgression lines (ILs) selected with SSRs from 2011-20 and their origin from the ‘Texas’ × ‘Earlygold’ (MB1.37) hybrid.

| **Year** | **fruits** | **seedlings** | **selected ILs** | **IL HET** | **IL HOM** | **T2E^1^** | **E2T^2^** | **E2TS1^3^** | **E3T^4^** |
| --- | --- | --- | --- | --- | --- | --- | --- | --- | --- |
| 2011-15 | 3551 | 836 | 137 | 109 | 28 | 8 | 28 | 101 | - |
| 2016 | 370 | 146 | 21 | 13 | 8 | - | - | 21 | - |
| 2017 | 1511 | 320 | 26 | 19 | 7 | - | - | 26 | - |
| 2018 | 782 | 324 | 25 | 14 | 11 | - | - | 25 | - |
| 2019 | 1853 | 418 | 62 | 42 | 20 | - | - | 53 | 9 |
| 2020 | 400 | 68 | 12 | 9 | 3 | - | - | 12 | - |
| Total | 8467 | 2112 | 283 | 206 | 77 | 8 | 28 | 238 | 9 |

^1^T2E: Second backcross generation to ‘Earlygold’ with ‘Texas’ cytoplasm; ^2^E2T Second backcross generation to ‘Earlygold’ with ‘Earlygold’ cytoplasm; ^3^E2TS1: selfed progeny of E2T; ^4^E3T: third backcross generation to ‘Earlygold’

**Supplementary Table 2.** Genotypes of the E2T set of 37 lines with two and three almond (‘Texas’) introgressions in heterozygosity on the peach (‘Earlygold’) background for 113 SSRs. The lines with three introgressions are marked with asterisks (*), unknown data (-). Linkage groups are from G1 to G8. Genotypes are H= heterozygous peach-almond; A=both alleles from peach.

**Supplementary Table 2.** (Continued)

**Supplementary Table 3.** Characteristics of the set of introgression lines (ILs) of almond fragments in the peach background in heterozygosis selected using the 18k SNP chip.

| **Line name** | **Initial code** | **Origin^1^** | **cs** | **Beginning of introgression (bp)** | **Marker in the proximal extreme** | **End of introgression (bp)** | **Marker in the distal extreme** | **Size (bp) of introgression** | **Age ^2^** | **Fruit available^3^** | **Comments** |
| --- | --- | --- | --- | --- | --- | --- | --- | --- | --- | --- | --- |
| PAILE1-0022 | E2T-022-13 | E2T‡ | 1 | 367,055 | Peach_AO_0000794 | 22,241,226 | Peach_AO_0070852 | 21,874,171 | 6 | Yes | - |
| PAILE1-0037* | 51P19-05 | E2TS1 | 1 | 367,055 | Peach_AO_0000794 | 36,948,923 | Peach_AO_0109269 | 36,581,868 | 1 | No | More than one fragment |
| PAILE1-2348 | 11P15-04 | E2T‡ | 1 | 23,494,321 | Peach_AO_0074055 | 47,719,653 | Peach_AO_0138791 | 24,225,332 | 5 | Yes | - |
| PAILE1-3448 | 21P15-39 | E2TS1 | 1 | 33,759,798 | Peach_AO_0101543 | 47,719,653 | Peach_AO_0138791 | 13,959,855 | 5 | Yes | - |
| PAILE2-0119 | 19P15-95 | E2TS1 | 2 | 1,349,947 | SNP_IGA_230270 | 19,091,424 | Peach_AO_0277409 | 17,741,477 | 5 | Yes | - |
| PAILE2-0125 | 19P15-58 | E2TS1 | 2 | 1,349,947 | SNP_IGA_230270 | 24,822,389 | Peach_AO_0295724 | 23,472,442 | 5 | Yes | - |
| PAILE2-0130a | 56P19-18 | E2TS1 | 2 | 1,349,947 | SNP_IGA_230270 | 30,092,550 | Peach_AO_0308231 | 28,742,603 | 1 | No | - |
| PAILE2-0130b | 56P19-23 | E2TS1 | 2 | 1,349,947 | SNP_IGA_230270 | 30,092,550 | Peach_AO_0308231 | 28,742,603 | 1 | No | - |
| PAILE2-1625 | 19P15-65 | E2TS1 | 2 | 16,477,856 | Peach_AO_0266461 | 24,822,389 | Peach_AO_0295724 | 8,344,533 | 5 | Yes | - |
| PAILE2-1925 | 19P15-04 | E2TS1 | 2 | 19,128,706 | Peach_AO_0277526 | 24,822,389 | Peach_AO_0295724 | 5,693,683 | 5 | Yes | - |
| PAILE2-2630 | 55P19-25 | E2TS1 | 2 | 25,893,886 | Peach_AO_0299018 | 30,092,550 | Peach_AO_0308231 | 4,198,664 | 1 | No | Small fragment |
| PAILE3-0006 | 19P15-77 | E2TS1 | 3 | 199,920 | Peach_AO_0309082 | 6,088,325 | Peach_AO_0329942 | 5,888,405 | 5 | Yes | - |
| PAILE3-0019 | 13P15-06 | E2T‡ | 3 | 199,920 | Peach_AO_0309082 | 19,025,208 | Peach_AO_0380211 | 18,825,288 | 5 | Yes | - |
| PAILE3-0020 | 01P19-10 | E3T | 3 | 199,920 | Peach_AO_0309082 | 20,039,043 | Peach_AO_0383420 | 19,839,123 | 1 | No | - |
| PAILE3-0026 | 01P19-02 | E3T | 3 | 199,920 | Peach_AO_0309082 | 26,417,128 | Peach_AO_0403611 | 26,217,208 | 1 | No | - |
| PAILE3-1827 | 04P20-01 | E2TS1 | 3 | 18,221,847 | SNP_3_12878608 | 27,311,852 | Peach_AO_0405265 | 9,090,005 | < 1 | No | - |
| PAILE4-0004 | 02P20-01 | E2TS1 | 4 | 109,846 | Peach_AO_0405407 | 3,954,996 | Peach_AO_0422387 | 3,845,150 | < 1 | No | Small fragment |
| PAILE4-0226a | T2E-193-06 | T2E‡ | 4 | 1,559,815 | Peach_AO_0415788 | 25,439,465 | Peach_AO_0525706 | 23,879,650 | 6 | Yes | - |
| PAILE4-0226b | T2E-193-29 | T2E‡ | 4 | 1,559,815 | Peach_AO_0415788 | 25,439,465 | Peach_AO_0525706 | 23,879,650 | 6 | Yes | - |

^1^ Origin: generation where the line has been selected; ‡plants that were grafted on ‘Garnem’ rootstock ^2^ Age: years from transplanting till 2021; ^3^ Trees producing fruit in 2021.

**Supplementary Table 3. (**Continued)

| **Line name** | **Initial code** | **Origin^1^** | **cs** | **Beginning of introgression (bp)** | **Marker in the proximal extreme** | **End of introgression (bp)** | **Marker in the distal extreme** | **Size (bp) of introgression** | **Age ^2^** | **Fruit available^3^** | **Comments** |
| --- | --- | --- | --- | --- | --- | --- | --- | --- | --- | --- | --- |
| PAILE4-0526 | T2E-193-19 | T2E‡ | 4 | 4,996,274 | Peach_AO_0425793 | 25,439,465 | Peach_AO_0525706 | 20,443,191 | 6 | Yes | - |
| PAILE5-0014 | 11P15-13 | E2T‡ | 5 | 277,967 | Peach_AO_0526579 | 13,961,772 | SNP_IGA_598118 | 13,683,805 | 5 | Yes | - |
| PAILE5-1319a | 61P19-09 | E2TS1 | 5 | 12,552,668 | Pp05_12552668 | 17,034,882 | Peach_AO_0590939 | 4,482,214 | 1 | No | - |
| PAILE5-1319b | 67P19-09 | E2TS1 | 5 | 12,552,668 | Pp05_12552668 | 17,034,882 | Peach_AO_0590939 | 4,482,214 | 1 | No | - |
| PAILE6-0008a | 30P18-31 | E2TS1 | 6 | 90,829 | Peach_AO_0592816 | 8,478,666 | SNP_IGA_631014 | 8,387,837 | 2 | No | - |
| PAILE6-0008b | 28P15-21 | E2TS1 | 6 | 90,829 | Peach_AO_0592816 | 8,478,666 | SNP_IGA_631014 | 8,387,837 | 2 | No | - |
| PAILE6-0009 | E2T-003-04 | E2T‡ | 6 | 90,829 | Peach_AO_0592816 | 9,281,763 | Peach_AO_0617940 | 9,190,934 | 6 | Yes | - |
| PAILE6-0020 | 30P18-18 | E2TS1 | 6 | 90,829 | Peach_AO_0592816 | 19,790,349 | Peach_AO_0659816 | 19,699,520 | 2 | No | - |
| PAILE6-0031a | 28P15-30 | E2TS1 | 6 | 90,829 | Peach_AO_0592816 | 30,560,829 | Peach_AO_0692360 | 30,470,000 | 5 | Yes | - |
| PAILE6-0031b | T2E-304-06 | T2E‡ | 6 | 90,829 | Peach_AO_0592816 | 30,560,829 | Peach_AO_0692360 | 30,470,000 | 6 | Yes | - |
| PAILE6-0031c | 04P17-01 | E2TS1 | 6 | 90,829 | Peach_AO_0592816 | 30,560,829 | Peach_AO_0692360 | 30,470,000 | 3 | No | - |
| PAILE6-0031d | 30P18-10 | E2TS1 | 6 | 90,829 | Peach_AO_0592816 | 30,560,829 | Peach_AO_0692360 | 30,470,000 | 2 | No | - |
| PAILE6-0831 | 09P15-04 | E2T‡ | 6 | 7,932,882 | SNP_IGA_629062 | 30,560,829 | Peach_AO_0692360 | 22,627,947 | 5 | Yes | - |
| PAILE7-0011 | 03P19-03 | E3T | 7 | 209,533 | Peach_AO_0693189 | 11,012,670 | SNP_IGA_762094 | 10,803,137 | 1 | No | - |
| PAILE7-0020 | 03P19-06 | E3T | 7 | 209,533 | Peach_AO_0693189 | 19,626,488 | Peach_AO_0772754 | 19,416,955 | 1 | No | - |
| PAILE7-0022a | 72P19-04 | E2TS1 | 7 | 209,533 | Peach_AO_0693189 | 21,814,037 | Peach_AO_0777606 | 21,604,504 | 1 | No | - |
| PAILE7-0022b | 01P19-05 | E3T | 7 | 209,533 | Peach_AO_0693189 | 22,231,288 | SNP_IGA_792877 | 22,021,755 | 1 | No | - |
| PAILE8-0020 | 31P18-01 | E2TS1 | 8 | 47,906 | Peach_AO_0778829 | 19,513,212 | Peach_AO_0867586 | 19,465,306 | 2 | No | - |
| PAILE8-0516 | 53P17-03 | E2TS1 | 8 | 5,159,866 | Peach_AO_0811535 | 16,340,573 | SNP_IGA_870207 | 11,180,707 | 3 | Yes | - |
| PAILE8-1623 | 24P15-27 | E2TS1 | 8 | 16,188,664 | Peach_AO_0855225 | 22,462,250 | Peach_AO_0875402 | 6,273,586 | 5 | Yes | - |

**Supplementary Table 4.** Characteristics of the set of introgression lines (ILs) of almond fragments in the peach background in homozygosis selected using the 18k SNP chip. All ILs selected in the E2TS1 generation.

| **Line name** | **Initial code** | **cs** | **Beginning of introgression (bp)** | **Marker in the proximal extreme** | **End of introgression (bp)** | **Marker in the distal extreme** | **Size (bp) of introgression** | **Age ^1^** | **Fruit available^2^** | **Comments** |
| --- | --- | --- | --- | --- | --- | --- | --- | --- | --- | --- |
| PAILO1-1131 | 75P19-03 | 1 | 11,452,860 | Peach_AO_0032086 | 31,554,767 | Peach_AO_0096835 | 20,101,907 | 1 | No | - |
| PAILO1-1148 | 57P18-44 | 1 | 11,452,860 | Peach_AO_0032086 | 47,719,653 | Peach_AO_0138791 | 36,266,793 | 2 | No | - |
| PAILO1-3148 | 30P18-03 | 1 | 30,835,956 | Peach_AO_0095680 | 47,719,653 | Peach_AO_0138791 | 16,883,697 | 2 | No | - |
| PAILO2-0123 | 06P17-15 | 2 | 1,349,947 | SNP_IGA_230270 | 22,838,257 | Peach_AO_0290341 | 21,488,310 | 3 | No | - |
| PAILO2-0125a | 19P15-50 | 2 | 1,349,947 | SNP_IGA_230270 | 24,822,389 | Peach_AO_0295724 | 23,472,442 | 5 | No | - |
| PAILO2-0125b | 19P15-120 | 2 | 1,349,947 | SNP_IGA_230270 | 24,822,389 | Peach_AO_0295724 | 23,472,442 | 5 | No | - |
| PAILO2-0130* | 37P18-44 | 2 | 1,349,947 | SNP_IGA_230270 | 30,092,550 | Peach_AO_0308231 | 28,742,603 | 2 | No | More than one fragment |
| PAILO2-0625 | 19P15-116 | 2 | 5,591,372 | SNP_IGA_182333 | 24,822,389 | Peach_AO_0295724 | 19,231,017 | 5 | No | - |
| PAILO3-0006 | 19P15-74 | 3 | 199,920 | Peach_AO_0309082 | 6,088,325 | Peach_AO_0329942 | 5,888,405 | 5 | Yes | - |
| PAILO3-0026 | 02P20-14 | 3 | 199,920 | Peach_AO_0309082 | 26,417,128 | Peach_AO_0403611 | 26,217,208 | < 1 | No | - |
| PAILO4-1526a | 28P15-34 | 4 | 14,698,356 | Peach_AO_0459684 | 25,439,465 | Peach_AO_0525706 | 10,741,109 | 5 | Yes | - |
| PAILO4-1526b | 28P15-46 | 4 | 14,698,356 | Peach_AO_0459684 | 25,439,465 | Peach_AO_0525706 | 10,741,109 | 5 | Yes | - |
| PAILO5-0014 | 16P17-02 | 5 | 277,967 | Peach_AO_0526579 | 14,038,734 | Peach_AO_0584194 | 13,760,767 | 3 | No | - |
| PAILO5-0419 | 09P17-01 | 5 | 3,993,107 | Peach_AO_0548234 | 17,034,882 | Peach_AO_0590939 | 13,041,775 | 3 | No | - |
| PAILO5-1219 | 67P19-03 | 5 | 12,477,386 | SNP_IGA_594745 | 17,034,882 | Peach_AO_0590939 | 4,557,496 | 1 | No | - |
| PAILO6-0005 | 20P15-66 | 6 | 90,829 | Peach_AO_0592816 | 5,435,066 | SNP_IGA_621556 | 5,344,237 | 5 | Yes | Small fragment |

^1^ Age: years from transplanting till 2021; ^2^ Trees producing fruit in 2021

**Supplementary Table 4.** (Continued)

| **Line name** | **Initial code** | **cs** | **Beginning of introgression (bp)** | **Marker in the proximal extreme** | **End of introgression (bp)** | **Marker in the distal extreme** | **Size (bp) of introgression** | **Age^1^** | **Fruit available^2^** | **Comments** |
| --- | --- | --- | --- | --- | --- | --- | --- | --- | --- | --- |
| PAILO6-0008 | 20P15-26 | 6 | 90,829 | Peach_AO_0592816 | 8,478,666 | SNP_IGA_631014 | 8,387,837 | 5 | Yes | - |
| PAILO6-0012 | 28P18-13 | 6 | 90,829 | Peach_AO_0592816 | 11,579,097 | Peach_AO_0626234 | 11,488,268 | 2 | No | - |
| PAILO6-0022 | 06P17-19 | 6 | 90,829 | Peach_AO_0592816 | 21,789,663 | Peach_AO_0665354 | 21,698,834 | 3 | No | - |
| PAILO6-0023 | 50P19-24 | 6 | 90,829 | Peach_AO_0592816 | 22,842,917 | SNP_IGA_677625 | 22,752,088 | 1 | No | - |
| PAILO6-0026 | 37P18-13 | 6 | 90,829 | Peach_AO_0592816 | 26,493,790 | SNP_IGA_688827 | 26,402,961 | 2 | No | - |
| PAILO6-0308 | 20P15-45 | 6 | 3,445,617 | Peach_AO_0601606 | 8,478,666 | SNP_IGA_631014 | 5,033,049 | 5 | Yes | Small fragment |
| PAILO6-0722 | 34P18-05 | 6 | 7,073,013 | Peach_AO_0611109 | 21,789,663 | Peach_AO_0665354 | 14,716,650 | 2 | No | - |
| PAILO7-0020 | 20P17-06 | 7 | 209,533 | Peach_AO_0693189 | 19,601,766 | Peach_AO_0772632 | 19,392,233 | 3 | No | - |
| PAILO7-2022 | 28P15-65 | 7 | 20,209,201 | Peach_AO_0774300 | 22,231,288 | SNP_IGA_792877 | 2,022,087 | 5 | Yes | Small fragment |
| PAILO8-0012 | 73P19-03 | 8 | 47,906 | Peach_AO_0778829 | 11,802,085 | Peach_AO_0839772 | 11,754,179 | 1 | No | - |
| PAILO8-0015 | 22P17-04 | 8 | 47,906 | Peach_AO_0778829 | 15,286,511 | Peach_AO_0852224 | 15,238,605 | 3 | Yes | - |
| PAILO8-1018* | 72P19-22 | 8 | 9,882,943 | Peach_AO_0831487 | 18,415,523 | Peach_AO_0863859 | 8,532,580 | 1 | No | More than one fragment |

**Supplementary Table 5.** Phenotypic data of the IL collections in homozygosis and heterozygosis. For juiciness (*Jui*): J = Juicy; NJ= non-juicy; for flesh color (*DBF2*): Y= yellow flesh; R=eed flesh; for resistance to powdery mildew (*Vr3*): S= susceptible; R= resistant; for maturity date (*MD*): Julian days until fruit maturity. Quantitative traits are FW (fruit weight), SSC (soluble solid content), TA (titratable acidity) and PL (petiole length). Data presented are the mean of three samples in all cases except for those with a ^1^ that are based on six samples. P with a subindex with the acronym of the trait indicates the probability of the Dunnett’s test for the comparison with ‘Earlygold’ (E) or with the rest of the IL collection except ILs with overlapping introgressions (IL): * P≤0.05; ** P≤0.01; *** P≤0.001, ns = non-significant.

|  | **Qualitative** | | | |  | **Quantitative** | | | | | | | | | | | |
| --- | --- | --- | --- | --- | --- | --- | --- | --- | --- | --- | --- | --- | --- | --- | --- | --- | --- |
|  | ***Jui*** | ***DBF2*** | ***Vr3*** | ***MD*** |  | **FW** | **P_FW_ (E)** | **P_FW_ (ILs)** | **SSC** | **P_SSC_ (E)** | **P_SSC_ (ILs)** | **TA** | **P_TA_ (E)** | **P_TA_ (ILs)** | **Petiole length** | **P_PL_ (E)** | **P_PL_ (ILs)** |
| Earlygold | J | Y | S | 158 |  | 82.67 | ns | ns | 10.23 | ns | ns | 3.91 | ns | ns | 0.98^1^ | ns | ns |
| PAILE1-0022 | J | Y | S | 155 |  | 69.67 | ns | * | 8.43 | ns | ns | 2.89 | ns | ns | 1.13 | ns | ns |
| PAILE1-0037* | - | - | S | - |  | - | - | - | - | - | - | - | - | - | 0.57 | ns | * |
| PAILE1-2348 | NJ | R | S | 165 |  | 106.00 | ** | ns | 10.10 | ns | ns | 4.15 | ns | ns | 1.10 | ns | ns |
| PAILE1-3448 | NJ | R | S | 194 |  | 104.33 | ** | ns | 10.70 | ns | ns | 4.50 | ns | ns | 1.03 | ns | ns |
| PAILE2-0119 | J | Y | R | 179 |  | 114.00 | *** | ns | 12.03 | ns | ns | 3.13 | ns | ns | 1.13 | ns | ns |
| PAILE2-0125 | J | Y | R | 179 |  | 115.67 | *** | ns | 12.40 | ns | ns | 4.80 | ns | ns | 1.03 | ns | ns |
| PAILE2-0130a | - | - | R | - |  | - | - | - | - | - | - | - | - | - | 0.70 | ns | ns |
| PAILE2-0130b | - | - | R | - |  | - | - | - | - | - | - | - | - | - | 0.63 | ns | ns |
| PAILE2-1625 | J | Y | R | 179 |  | 119.33 | *** | ns | 12.03 | ns | ns | 3.11 | ns | ns | 1.07 | ns | ns |
| PAILE2-1925 | J | Y | S | 173 |  | 111.33 | *** | ns | 11.93 | ns | ns | 3.92 | ns | ns | 0.97 | ns | ns |
| PAILE2-2630 | - | - | S | - |  | - | - | - | - | - | - | - | - | - | 0.50 | ** | ** |
| PAILE3-0006 | J | Y | S | 179 |  | 111.67 | *** | ns | 9.00 | ns | ns | 2.84 | ns | ns | 0.90 | ns | ns |
| PAILE3-0019 | J | Y | S | 173 |  | 123.00 | *** | ns | 9.13 | ns | ns | 5.19 | ns | ** | 0.83 | ns | ns |
| PAILE3-0020 | - | - | S | - |  | - | - | - | - | - | - | - | - | - | 0.77 | ns | ns |
| PAILE3-0026 | - | - | S | - |  | - | - | - | - | - | - | - | - | - | 0.67 | ns | ns |
| PAILE3-1827 | - | - | - |  |  | - | - | - | - | - | - | - | - | - | - | - | - |
| PAILE4-0004 | - | - | - |  |  | - | - | - | - | - | - | - | - | - | - | - | - |
| PAILE4-0226a | J | Y | S | 179 |  | 84.67 | ns | ns | 11.83 | ns | ns | 4.75 | ns | ns | 1.03 | ns | ns |

**Supplementary Table 5.** (Continued)

|  | **Qualitative** | | | |  | **Quantitative** | | | | | | | | | | | |
| --- | --- | --- | --- | --- | --- | --- | --- | --- | --- | --- | --- | --- | --- | --- | --- | --- | --- |
|  | ***Jui*** | ***DBF2*** | ***Vr3*** | ***MD*** |  | **FW** | **P_FW_ (E)** | **P_FW_ (ILs)** | **SSC** | **P_SSC_ (E)** | **P_SSC_ (ILs)** | **TA** | **P_TA_ (E)** | **P_TA_ (ILs)** | **Petiole length** | **P_PL_ (E)** | **P_PL_ (ILs)** |
| PAILE4-0226b | J | Y | S | 214 |  | 71.00 | ns | * | - | - | - | - | - | - | 0.87 | ns | ns |
| PAILE4-0526 | J | Y | S | 214 |  | 102.67 | * | ns | - | - | - | - | - | - | 1.10 | ns | ns |
| PAILE5-0014 | J | Y | S | 165 |  | 106.67 | ** | ns | 9.90 | ns | ns | 3.16 | ns | ns | 1.07 | ns | ns |
| PAILE5-1319a | - | - | S | - |  | - | - | - | - | - | - | - | - | - | 0.83 | ns | ns |
| PAILE5-1319b | - | - | S | - |  | - | - | - | - | - | - | - | - | - | 0.63 | ns | ns |
| PAILE6-0008a | - | - | S | - |  | - | - | - | - | - | - | - | - | - | 0.73 | ns | ns |
| PAILE6-0008b | J | Y | S | 161 |  | 101.00 | * | ns | 10.23 | ns | ns | 2.54 | * | * | 1.13 | ns | ns |
| PAILE6-0009 | J | Y | S | 158 |  | 57.00 | *** | *** | 6.77 | ** | *** | 5.35 | ns | ** | 0.77 | ns | ns |
| PAILE6-0020 | - | - | S | - |  | - | - | - | - | - | - | - | - | - | 1.33 | ns | * |
| PAILE6-0031a | J | Y | S | 161 |  | 93.67 | ns | ns | 9.97 | ns | ns | 3.43 | ns | ns | 1.00 | ns | ns |
| PAILE6-0031b | J | Y | S | 145 |  | 60.67 | ** | *** | 7.10 | * | *** | 2.71 | ns | * | 1.07 | ns | ns |
| PAILE6-0031c | - | - | S | - |  | - | - | - | - | - | - | - | - | - | - | - | - |
| PAILE6-0031d | - | - | S | - |  | - | - | - | - | - | - | - | - | - | 0.97 | ns | ns |
| PAILE6-0831 | J | Y | S | 165 |  | 106.00 | ** | ns | 13.83 | ** | * | 4.33 | ns | ns | 1.00 | ns | ns |
| PAILE7-0011 | - | - | S | - |  | - | - | - | - | - | - | - | - | - | 0.83 | ns | ns |
| PAILE7-0020 | - | - | S | - |  | - | - | - | - | - | - | - | - | - | 0.70 | ns | ns |
| PAILE7-0022a | - | - | S | - |  | - | - | - | - | - | - | - | - | - | 1.07 | ns | ns |
| PAILE7-0022b | - | - | S | - |  | - | - | - | - | - | - | - | - | - | 0.77 | ns | ns |
| PAILE8-0020 | - | - | - | - |  | - | - | - | - | - | - | - | - | - | - | - | - |
| PAILE8-0516 | J | Y | S | 161 |  | 97.33 | ns | ns | 13.30 | * | ns | 3.66 | ns | ns | 1.67^1^ | *** | *** |
| PAILE8-1623 | J | Y | S | 161 |  | 109.33 | *** | ns | 11.43 | ns | ns | 3.38 | ns | ns | 1.20 | ns | ns |
| PAILO1-1131 | - | - | S | - |  | - | - | - | - | - | - | - | - | - | - | - | - |

**Supplementary Table 5.** (Continued)

|  | **Qualitative** | | | |  | **Quantitative** | | | | | | | | | | | |
| --- | --- | --- | --- | --- | --- | --- | --- | --- | --- | --- | --- | --- | --- | --- | --- | --- | --- |
|  | ***Jui*** | ***DBF2*** | ***Vr3*** | ***MD*** |  | **FW** | **P_FW_ (E)** | **P_FW_ (ILs)** | **SSC** | **P_SSC_ (E)** | **P_SSC_ (ILs)** | **TA** | **P_TA_ (E)** | **P_TA_ (ILs)** | **Petiole length** | **P_PL_ (E)** | **P_PL_ (ILs)** |
| PAILO1-1148 | - | - | S | - |  | - | - | - | - | - | - | - | - | - | 0.53 | * | ** |
| PAILO1-3148 | - | - | S | - |  | - | - | - | - | - | - | - | - | - | 0.60 | * | * |
| PAILO2-0123 | - | - | R | - |  | - | - | - | - | - | - | - | - | - | 0.80 | ns | ns |
| PAILO2-0125a | - | - | R | - |  | - | - | - | - | - | - | - | - | - | 1.13 | ns | ns |
| PAILO2-0125b | - | - | R | - |  | - | - | - | - | - | - | - | - | - | 0.90 | ns | ns |
| PAILO2-0130* | - | - | R | - |  | - | - | - | - | - | - | - | - | - | - | - | - |
| PAILO2-0625 | - | - | R | - |  | - | - | - | - | - | - | - | - | - | 0.97 | ns | ns |
| PAILO3-0006 | J | Y | S | 168 |  | 102.33 | * | ns | 11.23 | ns | ns | 3.78 | ns | ns | 0.97 | ns | ns |
| PAILO3-0026 | - | - | - | - |  | - | - | - | - | - | - | - | - | - | - | - | - |
| PAILO4-1526a | J | Y | S | 161 |  | 80.00 | ns | ns | 9.60 | ns | ns | 2.84 | ns | ns | 0.70 | ns | ns |
| PAILO4-1526b | J | Y | S | 165 |  | 83.33 | ns | ns | 10.50 | ns | ns | 2.86 | ns | ns | 1.13 | ns | ns |
| PAILO5-0014 | - | - | S | - |  | - | - | - | - | - | - | - | - | - | 0.87 | ns | ns |
| PAILO5-0419 | - | - | S | - |  | - | - | - | - | - | - | - | - | - | 0.97 | ns | ns |
| PAILO5-1219 | - | - | S | - |  | - | - | - | - | - | - | - | - | - | - | - | - |
| PAILO6-0005 | J | Y | S | 187 |  | 144.33 | *** | *** | 11.63 | ns | ns | 3.55 | ns | ns | 0.93 | ns | ns |
| PAILO6-0008 | J | Y | S | 168 |  | 148.33 | *** | *** | 13.20 | * | * | 4.63 | ns | ns | 1.07 | ns | ns |
| PAILO6-0012 | - | - | S | - |  | - | - | - | - | - | - | - | - | - | 0.80 | ns | ns |
| PAILO6-0022 | - | - | S | - |  | - | - | - | - | - | - | - | - | - | 1.10 | ns | ns |
| PAILO6-0023 | - | - | S | - |  | - | - | - | - | - | - | - | - | - | 0.70 | ns | ns |
| PAILO6-0026 | - | - | S | - |  | - | - | - | - | - | - | - | - | - | 0.67 | ns | ns |
| PAILO6-0308 | J | Y | S | 194 |  | 84.67 | ns | ns | 16.87 | *** | *** | 2.41 | ns | * | 0.73 | ns | ns |

**Supplementary Table 5.** (Continued)

|  | **Qualitative** | | | |  | **Quantitative** | | | | | | | | | | | |
| --- | --- | --- | --- | --- | --- | --- | --- | --- | --- | --- | --- | --- | --- | --- | --- | --- | --- |
|  | ***Jui*** | ***DBF2*** | ***Vr3*** | ***MD*** |  | **FW** | **P_FW_ (E)** | **P_FW_ (ILs)** | **SSC** | **P_SSC_ (E)** | **P_SSC_ (ILs)** | **TA** | **P_TA_ (E)** | **P_TA_ (ILs)** | **Petiole length** | **P_PL_ (E)** | **P_PL_ (ILs)** |
| PAILO6-0722 | - | - | S | - |  | - | - | - | - | - | - | - | - | - | 0.67^1^ | * | * |
| PAILO7-0020 | - | - | S | - |  | - | - | - | - | - | - | - | - | - | 1.00 | ns | ns |
| PAILO7-2022 | J | Y | S | 155 |  | 76.33 | ns | ns | 8.30 | ns | ns | 2.74 | ns | ns | 1.33 | ns | * |
| PAILO8-0012 | J | Y | S | 152 |  | - | - | - | - | - | - | - | - | - | 0.97 | ns | ns |
| PAILO8-0015 | - | - | S | - |  | - | - | - | - | - | - | - | - | - | 1.20 | ns | * |
| PAILO8-1018* | - | - | S | - |  | - | - | - | - | - | - | - | - | - | 1.40^1^ | ** | *** |

**Supplementary Table 6**. Summary of consistent QTLs for the quantitative characters studied in the IL collection, previously detected in the E×E map (‘Earlygold’ F2 population) as described in Kalluri et al.^23^ and in the T×E (F2 progeny of ‘Texas’ × ‘Earlygold’) and T1E [(‘Texas’ × ‘Earlygold’) × ‘Earlygold’ population], from Donoso et al.^20^ Trait category, map type, QTL names, LOD score of the maximum peak, linkage group, map position of the maximum peak, percentage of explained phenotypic variance (R^2^), additivity (a), dominance (d), d/a, gene action (GA).

| **Trait** | **Map** | **QTL/**  **gene name** | **LOD** | **G** | **Position (cM)** | **R^2^** | **a** | **d** | **d/a** | **GA^1^** |
| --- | --- | --- | --- | --- | --- | --- | --- | --- | --- | --- |
| Maturity date | E×E | qMD4 | 24.9 - 27.1 | 4 | 34.3-37.1 | 80.6 - 82.0 | 9.6 to 10.9 | -1.2 to - 5.2 | -0.1 to -0.5 | A/D |
|  | T×E | *MD* | - | 4 | 32.8 | - | - | - | - | coD |
|  | T1E | qMD4 | 4.5 - 11.9 | 4 | 31.6-40.6 | 32.9 - 57.9 | 17.7 - 40.0 | - | - | - |
| Fruit weight | E×E | qFW4 | 4.9 - 7.7 | 4 | 33.6-37.1 | 28.0 - 38.6 | 12.2 to 18.3 | -0.9 to - 6.4 | -0.4 to 0.2 | A |
|  | T×E | qFD4 | 2.9 - 8.7 | 4 | 31.7 - 36.9 | 35.8 - 77.5 | -10.7 to -15.7 | 24.8 - 33.9 | -0.9 to -3.1 | DP/U |
|  | T1E | qFW6 | 2.7 - 5.3 | 6 | 0.0 | 18.9 - 21.3 | 24.8 - 28.6 | - | - | - |
| Soluble solid content | E×E | qSSC4 | 8.8 - 9.5 | 4 | 36.4 - 37.1 | 44.3 - 47.4 | 1.2 to 1.6 | -0.5 to 0.2 | -0.3 to 0.1 | A |
| Titratable acidity | E×E | qTA6 | 3.0 - 6.4 | 6 | 48.1 - 55.6 | 17.4 - 34.9 | -0.7 to -0.9 | 0.1 to 0.2 | -0.1 to -0.3 | A |
| Petiole length | T×E, T1E | qPL5 | 2.7 - 4.8 | 5 | 12.9 - 28.3 | 8.9 - 28.1 | 15.3 - 29.2 | 5.7 - 10.1 | 0.2 - 0.5 | A/AD |
|  | T1E | qPL6 | 2.6 - 4.1 | 6 | 34.4 - 34.9 | 8.3 - 13.5 | 14.8 – 19.6 | - | - | - |
|  | T1E | qPL7 | 2.7 - 4.2 | 7 | 24.1 - 27.9 | 9.2 - 13.8 | 16.5-19.8 | - | - | - |
|  | T×E, T1E | qPL8 | 2.7 - 13.6 | 8 | 12.2 - 44.8 | 14.9 - 39.0 | 18.7-33.1 | 5.9 - 20.0 | 0.2 - 1.2 | DA/A |

^1^GA: Gene Action: A additivity, D dominance, AD partial dominance for almond allele, DA dominance for almond allele, DP dominance for the peach allele, coD codominance, and U = underdominance

**Supplementary Table 7.** Phenotypic data of the quantitative traits studied**:** FW (fruit weight), SSC (soluble solid content), TA (titratable acidity) and PL (petiole length). The numbers (1-6) correspond to the replicates.

|  | **FW** | | | **SSC** | | | **TA** | | | **PL** | | | | | |
| --- | --- | --- | --- | --- | --- | --- | --- | --- | --- | --- | --- | --- | --- | --- | --- |
|  | **1** | **2** | **3** | **1** | **2** | **3** | **1** | **2** | **3** | **1** | **2** | **3** | **4** | **5** | **6** |
| EG | 75 | 82 | 91 | 10.60 | 9.40 | 10.70 | 3.88 | 3.21 | 4.63 | 1.10 | 1.00 | 0.80 | 0.90 | 1.10 | 1.00 |
| PAILE1-0022 | 66 | 77 | 66 | 9.10 | 7.00 | 9.20 | 2.61 | 2.69 | 3.36 | 0.80 | 1.10 | 1.50 | - | - | - |
| PAILE1-0037* | - | - | - | - | - | - | - | - | - | 0.60 | 0.60 | 0.50 | - | - | - |
| PAILE1-2348 | 101 | 118 | 99 | 10.00 | 11.30 | 9.00 | 3.73 | 4.85 | 3.88 | 1.00 | 1.50 | 0.80 | - | - | - |
| PAILE1-3448 | 95 | 103 | 115 | 11.50 | 10.20 | 10.40 | 4.10 | 4.33 | 5.07 | 1.10 | 0.90 | 1.10 | - | - | - |
| PAILE2-0119 | 111 | 116 | 115 | 11.50 | 13.20 | 11.40 | 3.43 | 3.36 | 2.61 | 1.10 | 1.20 | 1.10 | - | - | - |
| PAILE2-0125 | 114 | 120 | 113 | 12.80 | 12.90 | 11.50 | 5.22 | 5.22 | 3.96 | 0.90 | 1.10 | 1.10 | - | - | - |
| PAILE2-0130a | - | - | - | - | - | - | - | - | - | 0.60 | 0.90 | 0.60 | - | - | - |
| PAILE2-0130b | - | - | - | - | - | - | - | - | - | 0.70 | 0.60 | 0.60 | - | - | - |
| PAILE2-1625 | 119 | 122 | 117 | 11.90 | 11.90 | 12.30 | 2.99 | 3.21 | 3.13 | 1.20 | 1.00 | 1.00 | - | - | - |
| PAILE2-1925 | 115 | 114 | 105 | 11.30 | 12.40 | 12.10 | 4.18 | 3.66 | - | 0.90 | 1.00 | 1.00 | - | - | - |
| PAILE2-2630 | - | - | - | - | - | - | - | - | - | 0.50 | 0.50 | 0.50 | - | - | - |
| PAILE3-0006 | 113 | 110 | 112 | 8.10 | 10.30 | 8.60 | 2.69 | 2.99 | 2.84 | 1.00 | 0.90 | 0.80 | - | - | - |
| PAILE3-0019 | 127 | 124 | 118 | 9.40 | 9.10 | 8.90 | 4.48 | 5.90 | - | 0.30 | 1.10 | 1.10 | - | - | - |
| PAILE3-0020 | - | - | - | - | - | - | - | - | - | 0.90 | 0.90 | 0.50 | - | - | - |
| PAILE3-0026 | - | - | - | - | - | - | - | - | - | 0.50 | 0.60 | 0.90 | - | - | - |
| PAILE4-0226a | 80 | 88 | 86 | 11.50 | 12.60 | 11.40 | 4.48 | 5.67 | 4.10 | 1.00 | 1.30 | 0.80 | - | - | - |
| PAILE4-0226b | 75 | 70 | 68 | - | - | - | - | - | - | 1.00 | 0.80 | 0.80 | - | - | - |
| PAILE4-0526 | 112 | 101 | 95 | - | - | - | - | - | - | 1.00 | 1.10 | 1.20 | - | - | - |
| PAILE5-0014 | 100 | 104 | 116 | 9.20 | 10.10 | 10.40 | 2.99 | 2.99 | 3.51 | 1.00 | 1.00 | 1.20 | - | - | - |
| PAILE5-1319a | - | - | - | - | - | - | - | - | - | 0.80 | 0.70 | 1.00 | - | - | - |
| PAILE5-1319b | - | - | - | - | - | - | - | - | - | 0.50 | 0.70 | 0.70 | - | - | - |
| PAILE6-0008a | - | - | - | - | - | - | - | - | - | 0.80 | 0.60 | 0.80 | - | - | - |
| PAILE6-0008b | 106 | 113 | 84 | 11.30 | 10.70 | 8.70 | 2.84 | 2.54 | 2.24 | 1.20 | 1.00 | 1.20 | - | - | - |
| PAILE6-0009 | 52 | 53 | 66 | 4.10 | 7.70 | 8.50 | 3.88 | 6.27 | 5.90 | 0.80 | 0.70 | 0.80 | - | - | - |

**Supplementary Table 7.** (Continued)

|  | **FW** | | | **SSC** | | | **TA** | | | **PL** | | | | | |
| --- | --- | --- | --- | --- | --- | --- | --- | --- | --- | --- | --- | --- | --- | --- | --- |
|  | **1** | **2** | **3** | **1** | **2** | **3** | **1** | **2** | **3** | **1** | **2** | **3** | **4** | **5** | **6** |
| PAILE6-0020 | - | - | - | - | - | - | - | - | - | 1.80 | 1.20 | 1.00 | - | - | - |
| PAILE6-0031a | 103 | 89 | 89 | 9.90 | 9.70 | 10.30 | 3.88 | 3.43 | 2.99 | 1.00 | 1.00 | 1.00 | - | - | - |
| PAILE6-0031b | 71 | 50 | 61 | 7.50 | 8.50 | 5.30 | 4.48 | 1.79 | 1.87 | 1.00 | 1.20 | 1.00 | - | - | - |
| PAILE6-0031d | - | - | - | - | - | - | - | - | - | 1.10 | 0.90 | 0.90 | - | - | - |
| PAILE6-0831 | 104 | 108 | 103 | 13.10 | 13.70 | 14.70 | 3.58 | 6.12 | 3.28 | 1.00 | 1.00 | 1.00 | - | - | - |
| PAILE7-0011 | - | - | - | - | - | - | - | - | - | 0.60 | 1.00 | 0.90 | - | - | - |
| PAILE7-0020 | - | - | - | - | - | - | - | - | - | 0.80 | 0.70 | 0.60 | - | - | - |
| PAILE7-0022a | - | - | - | - | - | - | - | - | - | 1.00 | 1.00 | 1.20 | - | - | - |
| PAILE7-0022b | - | - | - | - | - | - | - | - | - | 0.70 | 0.60 | 1.00 | - | - | - |
| PAILE8-0516 | 92 | 100 | 100 | 13.30 | 11.60 | 15.00 | 2.84 | 3.96 | 4.18 | 1.70 | 1.70 | 1.60 | 1.50 | 1.80 | 1.70 |
| PAILE8-1623 | 119 | 106 | 103 | 11.40 | 11.20 | 11.70 | 3.28 | 3.66 | 3.21 | 1.30 | 1.30 | 1.00 | - | - | - |
| PAILO1-1148 | - | - | - | - | - | - | - | - | - | 0.70 | 0.40 | 0.50 | - | - | - |
| PAILO1-3148 | - | - | - | - | - | - | - | - | - | 0.60 | 0.60 | 0.60 | - | - | - |
| PAILO2-0123 | - | - | - | - | - | - | - | - | - | 0.60 | 1.00 | 0.80 | - | - | - |
| PAILO2-0125a | - | - | - | - | - | - | - | - | - | 1.10 | 1.20 | 1.10 | - | - | - |
| PAILO2-0125b | - | - | - | - | - | - | - | - | - | 0.90 | 0.90 | 0.90 | - | - | - |
| PAILO2-0625 | - | - | - | - | - | - | - | - | - | 1.00 | 1.00 | 0.90 | - | - | - |
| PAILO3-0006 | 107 | 103 | 97 | 11.90 | 11.40 | 10.40 | 3.88 | 3.81 | 3.66 | 0.90 | 1.00 | 1.00 | - | - | - |
| PAILO4-1526a | 89 | 78 | 73 | 10.30 | 11.60 | 6.90 | 2.84 | 2.99 | 2.69 | 0.70 | 0.80 | 0.60 | - | - | - |
| PAILO4-1526b | 90 | 81 | 79 | 11.60 | 10.70 | 9.20 | 2.91 | 3.28 | 2.39 | 1.20 | 1.10 | 1.10 | - | - | - |
| PAILO5-0014 | - | - | - | - | - | - | - | - | - | 0.90 | 0.70 | 1.00 | - | - | - |
| PAILO5-0419 | - | - | - | - | - | - | - | - | - | 0.80 | 0.90 | 1.20 | - | - | - |
| PAILO6-0005 | 142 | 151 | 140 | 11.50 | 11.30 | 12.10 | 3.13 | 3.43 | 4.10 | 0.80 | 1.00 | 1.00 | - | - | - |
| PAILO6-0008 | 145 | 149 | 151 | 13.50 | 13.20 | 12.90 | 4.18 | 5.30 | 4.40 | 1.20 | 1.00 | 1.00 | - | - | - |
| PAILO6-0012 | - | - | - | - | - | - | - | - | - | 0.60 | 1.00 | 0.80 | - | - | - |
| PAILO6-0022 | - | - | - | - | - | - | - | - | - | 1.10 | 1.10 | 1.10 | - | - | - |

**Supplementary Table 7.** (Continued)

|  | **FW** | | | **SSC** | | | **TA** | | | **PL** | | | | | |
| --- | --- | --- | --- | --- | --- | --- | --- | --- | --- | --- | --- | --- | --- | --- | --- |
|  | **1** | **2** | **3** | **1** | **2** | **3** | **1** | **2** | **3** | **1** | **2** | **3** | **4** | **5** | **6** |
| PAILO6-0023 | - | - | - | - | - | - | - | - | - | 0.80 | 0.60 | 0.70 | - | - | - |
| PAILO6-0026 | - | - | - | - | - | - | - | - | - | 0.60 | 0.80 | 0.60 | - | - | - |
| PAILO6-0308 | 87 | 83 | 84 | 17.50 | 15.60 | 17.50 | 2.39 | 2.69 | 2.16 | 0.80 | 0.70 | 0.70 | - | - | - |
| PAILO6-0722 | - | - | - | - | - | - | - | - | - | 0.40 | 0.50 | 0.60 | 0.90 | 0.80 | 0.80 |
| PAILO7-0020 | - | - | - | - | - | - | - | - | - | 1.00 | 1.00 | 1.00 | - | - | - |
| PAILO7-2022 | 80 | 78 | 71 | 8.90 | 7.00 | 9.00 | 3.88 | 1.87 | 2.46 | 1.40 | 1.30 | 1.30 | - | - | - |
| PAILO8-0012 | - | - | - | - | - | - | - | - | - | 1.10 | 0.80 | 1.00 | - | - | - |
| PAILO8-0015 | - | - | - | - | - | - | - | - | - | 1.40 | 1.10 | 1.10 | - | - | - |
| PAILO8-1018* | - | - | - | - | - | - | - | - | - | 1.50 | 1.30 | 0.80 | 1.40 | 2.00 | 1.40 |

**Supplementary Figure 1.** Graphical genotype of the E2T set with two and three almond introgressions in heterozygosity based on 113 SSR markers and their genetic distance. Lines marked with asterisk (*) have three introgressions and the rest two introgressions each. Gray color corresponds to the peach ‘Earlygold’ background and red to the ‘Texas’ almond introgressions.

**
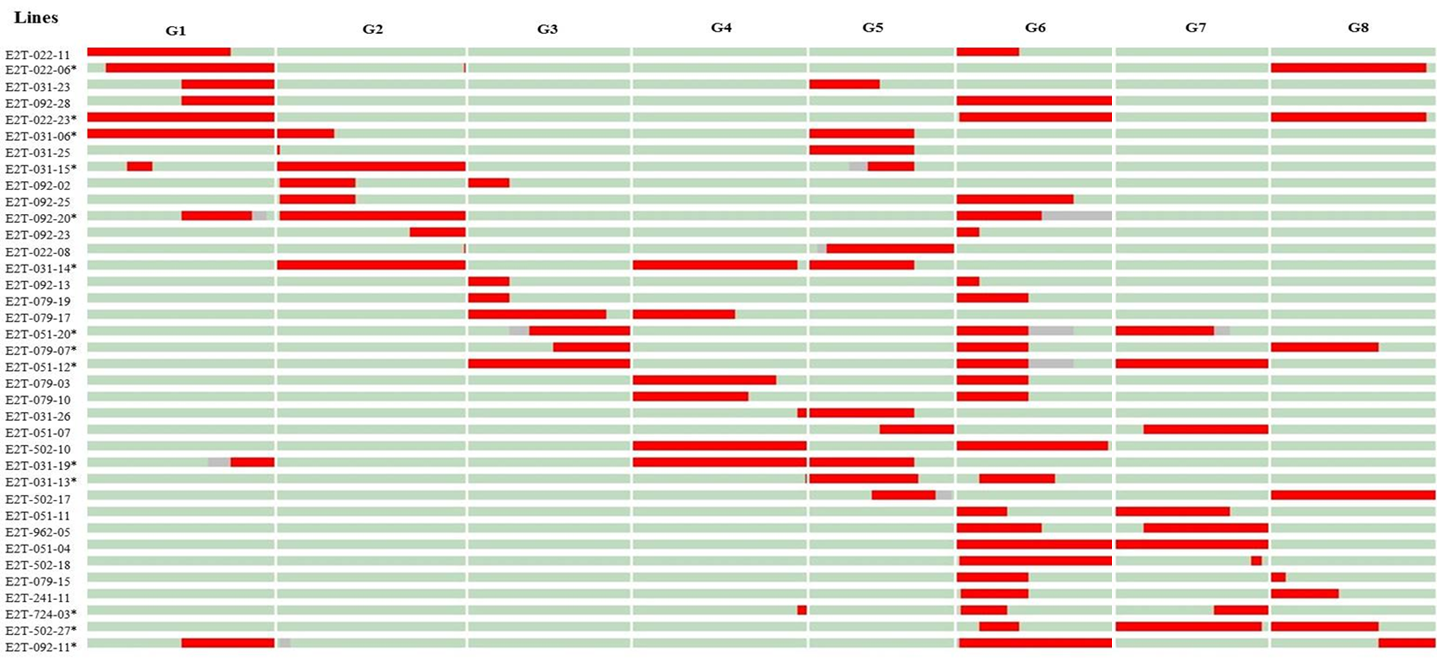
**

**Supplementary Figure 2.** Graphical representation of the 206 heterozygous ILs of almond fragments (red) in the peach background (grey) based on SSR markers and their genetic distance. G1 to G8 are the eight linkage groups of *Prunus*.

**
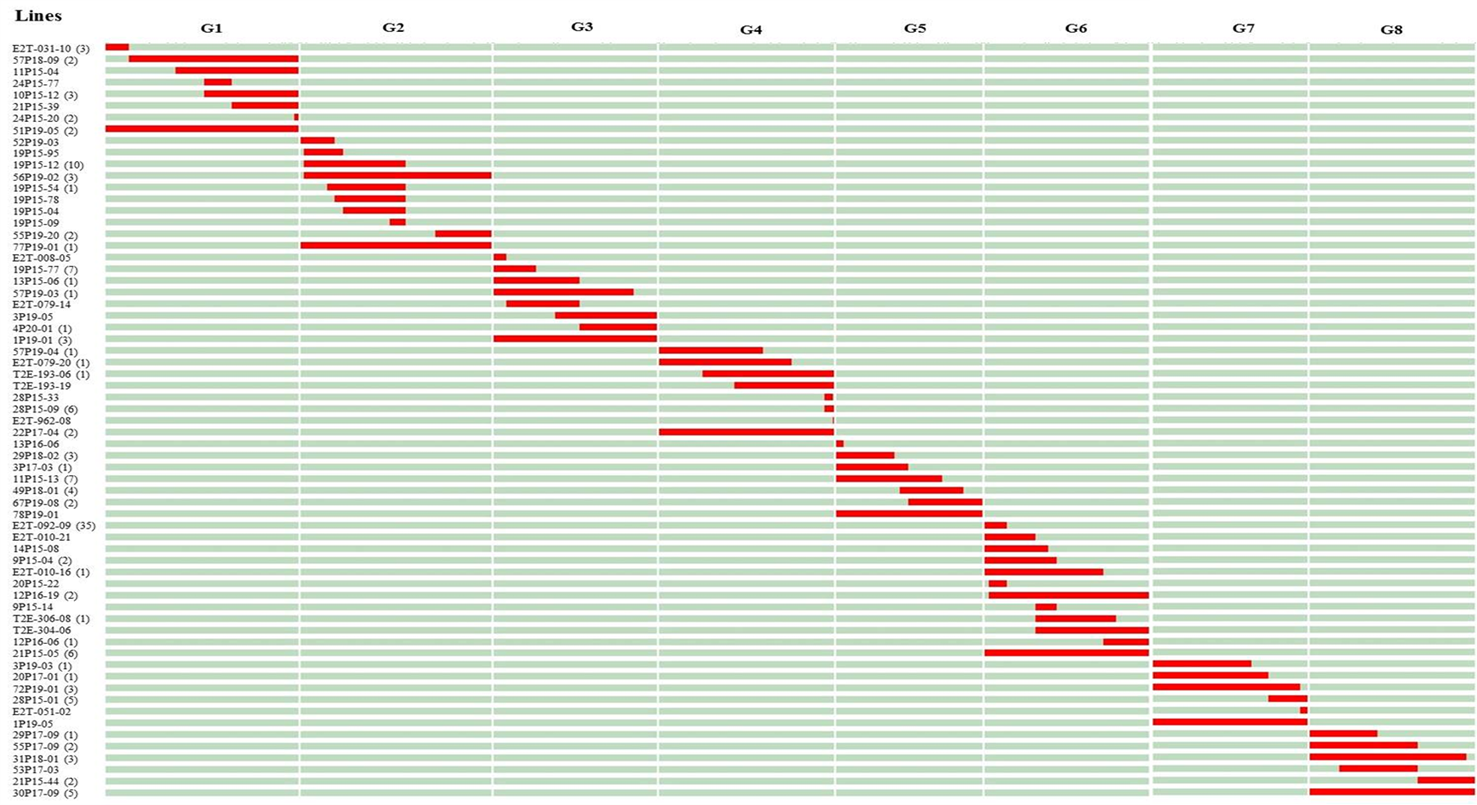
**

**Supplementary Figure 3.** Graphical representation of the 77 homozygous ILs of almond fragments (blue) in the peach background (grey) based on SSR markers and their genetic distance. Red fragments are heterozygous almond introgressions. G1 to G8 are the eight linkage groups of *Prunus*.

**
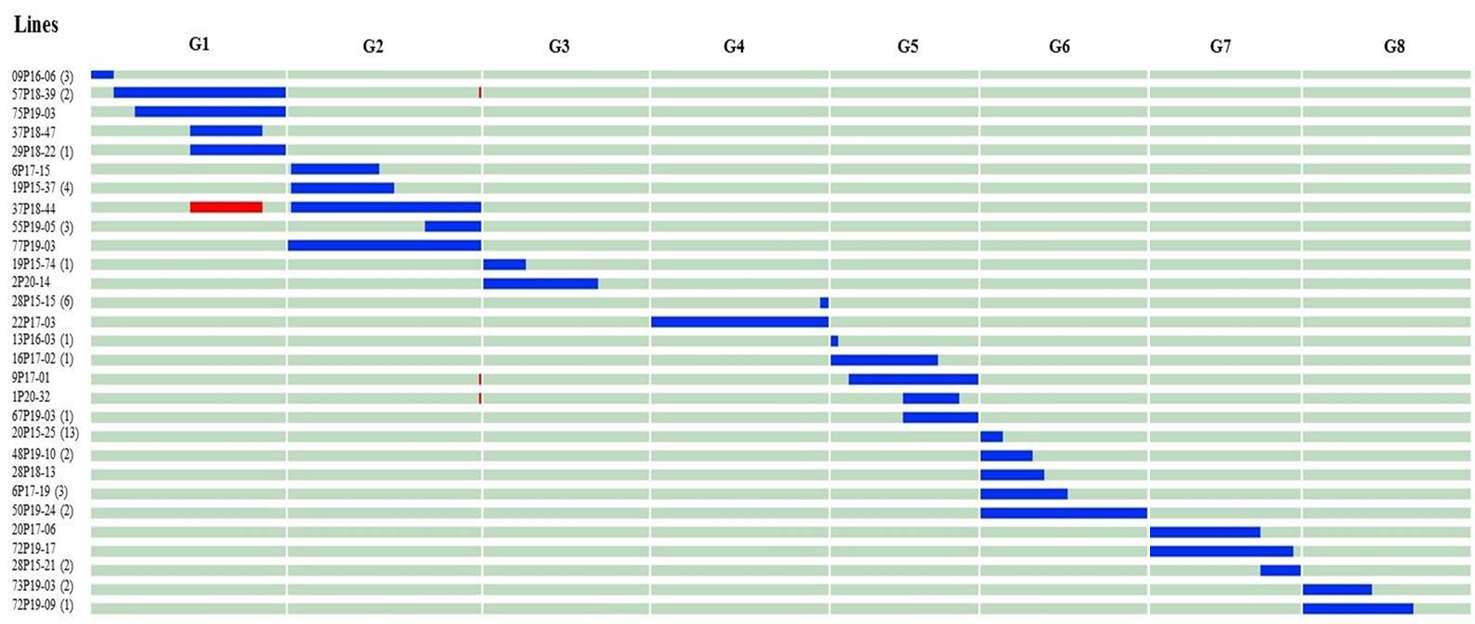
**
